# Efficacy and Safety of a New Resilient Hyaluronic Acid Filler in the Correction of Moderate-to-Severe Dynamic Perioral Rhytides: A 52-Week Prospective, Multicenter, Controlled, Randomized, Evaluator-Blinded Study

**DOI:** 10.1097/DSS.0000000000003238

**Published:** 2021-09-30

**Authors:** Hema Sundaram, Ava Shamban, Joel Schlessinger, Joely Kaufman-Janette, John H. Joseph, Mark Lupin, Zoe Draelos, Wayne Carey, Stacy Smith, Laura Eaton

**Affiliations:** * Dermatology, Cosmetic, and Laser Surgery Center, Rockville, Maryland and Fairfax, Virginia;; † ATS Clinical Research, Santa Monica, California;; ‡ Skin Specialists PC, Omaha, Nebraska;; § Skin Research Institute, LLC, Coral Gables, Florida;; ‖ Clinical Testing of Beverly Hills, Beverly Hills, California;; ¶ Cosmedica Laser Center, Victoria, Canada;; ** Dermatology Consulting Services, High Point, North Carolina;; †† Siena Medical Research Corporation, Montreal, Canada;; ‡‡ California Dermatology & Clinical Research Institute, Encinitas, California;; §§ UltaMed Corporation, Fort Lauderdale, Florida

## Abstract

The perioral region is highly mobile and subject to multifactorial changes during aging. Resilient Hyaluronic Acid Redensity (RHA_R_), an RHA filler, was developed with the aim of optimizing outcomes in dynamic facial areas.

**OBJECTIVE:**

This randomized, blinded, multicenter clinical study aimed to demonstrate superiority of RHA_R_ over no-treatment control for correction of moderate-to-severe dynamic perioral rhytides.

**MATERIALS AND METHODS:**

Blinded live evaluator assessments of efficacy included improvement in perioral rhytides severity using a proprietary scale (Perioral Rhytids Severity Rating Scale [PR-SRS]) and the Global Aesthetic Improvement Scale. Subjects self-assessed their results with FACE-Q, a validated patient-reported outcome measure, and satisfaction scales. Safety was monitored throughout the study based on common treatment responses (CTRs) and adverse events (AEs).

**RESULTS:**

The primary efficacy end point was achieved, with the treatment group showing statistically significant superiority over the control group at Week 8 (80.7% vs 7.8% responder rate by PR-SRS, *p* < .0001). Most patients (66%) were still responders at Week 52 (study completion). Most AEs were CTRs after perioral injection of a dermal filler, and none was a clinically significant treatment-related AE.

**CONCLUSION:**

Resilient Hyaluronic Acid Redensity is effective and safe for the correction of dynamic perioral rhytides in all Fitzpatrick phototypes, with marked durability.

## Abstract 2

Supplemental Digital Content is Available in the Text.

Perioral aging is highly individualized, comprising several distinct and simultaneous processes in the bone, and subcutaneous and cutaneous tissues that are driven by extrinsic and intrinsic factors.^1–4^

Manifestations of volume loss in the upper lip include decreased tissue elasticity, lip ptosis, decreased lip thickness relative to length, loss of lip volume, contour and vermilion border eversion, and skin textural changes.^2,5,6^ Fine vertical rhytides above the upper lip and under the lower lip are primarily related to repetitive movements, photoaging with thinning of the skin and subcutis,^7^ and resorption of underlying bone and dentition.

Risk factors for development of perioral rhytides include age, history of smoking, cumulative sun exposure, sex, and skin phototype. Greater innate photoprotection contributes to the decreased propensity of individuals with higher Fitzpatrick skin phototypes to develop perioral lines.^8^ Female patients tend to develop more and deeper perioral wrinkling, and more pronounced general manifestations of lip aging.^8,9^

The perioral region and lips are critical markers of youth, attractiveness, and beauty.^10^ Although injection of hyaluronic acid (HA) dermal filler into perioral lines is a common rejuvenation strategy, the structural and functional anatomy of this region, as well as its mobility, present significant challenges.

The investigational device for this study, Resilient Hyaluronic Acid Redensity (RHA_R_), belongs to a range of RHA fillers that are specifically designed to adapt to facial dynamics. Resilient hyaluronic acid fillers are crosslinked with 1,4-butanediol diglycidyl ether (BDDE) using a unique manufacturing process, the Preserved Network technology, that results in a greater preservation of HA chains, thus requiring a low degree of modification (MoD) to provide clinically relevant product properties.^11^ Resilient Hyaluronic Acid Redensity has a HA concentration of 15 mg/mL and a low MoD of 2%, as compared with 5% to 10% in most HA gels,^12^ translating into rheological properties adapted to treat fine lines and superficial wrinkles in mobile areas such as the perioral region.^13,14^

Biomechanical characterization of RHA fillers has demonstrated their resilient behavior, that is, improved capacity to recover their original shape and mechanical features after compression, stretching, or bending.^11,13^ Other investigators have reported homogeneous tissue integration of RHA fillers after intradermal injection.^14^

This study was conducted to demonstrate the superiority of RHA_R_ over no-treatment control for the correction of moderate-to-severe dynamic perioral rhytides, as assessed by a blinded live evaluator on the Perioral Rhytids Severity Rating Scale (PR-SRS; Figure 1) 8 weeks after treatment. The study also evaluated product safety and durability of the aesthetic improvement, up to 52 weeks after initial or touch-up injection.

**Figure 1.**
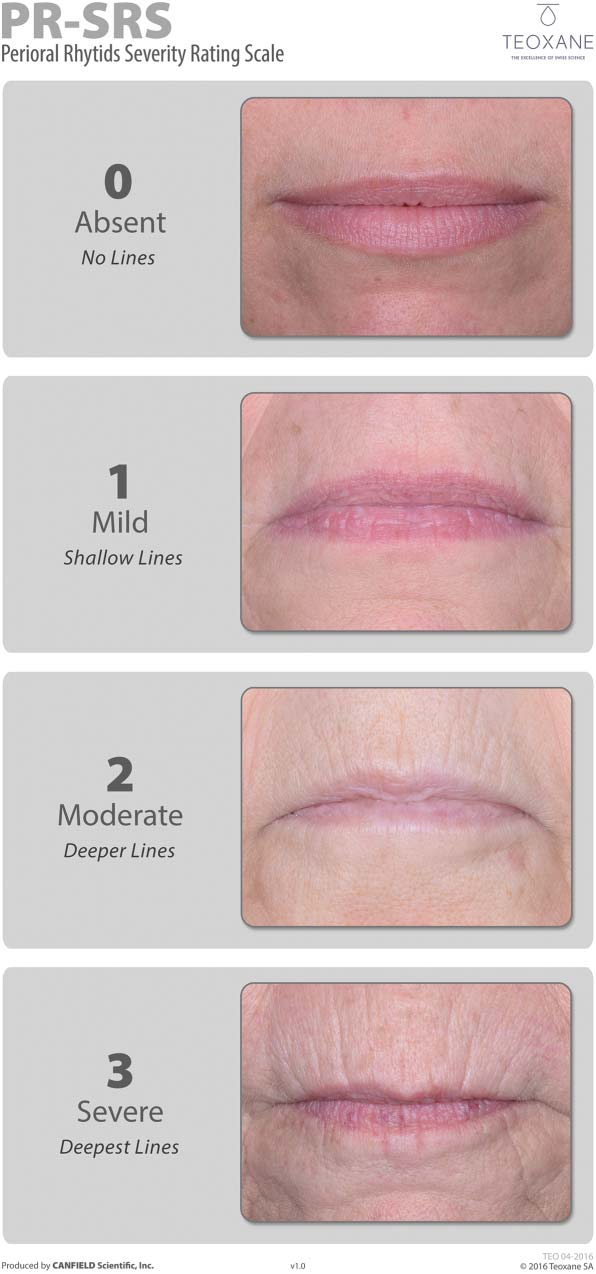
Perioral Rhytids Severity Rating Scale (PR-SRS). A proprietary 4-grade rating scale [0–3].

## Materials and Methods

### Trial Design and Population

This randomized, single-blinded, no-treatment control, multicenter, prospective clinical trial was conducted in accordance with the International Conference on Harmonization, Good Clinical Practice guidelines, Code of Federal Regulations, and the Declaration of Helsinki. The study received approval from an institutional review board and was registered on ClinicalTrials.gov (NCT03092219). All subjects gave informed consent before any study procedures being performed.

The target population for inclusion was adults 22 years of age and older, with moderate-to-severe perioral rhytides (Grade 2 or 3 on the 4-point scale, PR-SRS^15^). The study aimed to recruit at least 25% subjects with Fitzpatrick skin phototypes IV to VI. Considering the global population size, this was sufficient for detection of any adverse event (AE) with an incidence of 2.5% or more in these skin types.

Subjects who met all inclusion criteria and no exclusion criteria were randomized 3:1 to treatment with RHA_R_ (treatment group) or to no treatment (control group). Subjects in the control group commenced treatment with RHA_R_ at Week 8 after primary end point evaluation. They subsequently followed the same schedule of injection and evaluation as subjects in the treatment group (see **Supplemental Digital Content 1**, Figure S1, http://links.lww.com/DSS/A911, which demonstrates the study schematics).

### Treatment

Resilient Hyaluronic Acid Redensity (TEOXANE S.A., Geneva, Switzerland) is a dermal filler composed of 15 mg/mL high-molecular-weight, BDDE-crosslinked HA, and 0.3% lidocaine hydrochloride, in a physiologic phosphate buffer (pH 7.3). The device was injected using 30-gauge ½-inch needles. The treatment group received injections of RHA_R_ into the upper and lower perioral rhytides at Day 0 (Visit 1). Subjects were offered optional touch-up treatment 14 days later to provide optimal aesthetic correction. Subjects were eligible for repeat treatment at 12, 16, 24, or 36 weeks after their last treatment if their PR-SRS score had returned to its baseline value. All subjects were offered retreatment at the end of the study (Week 52).

Supplemental preinjection and postinjection pain control was at the investigator's discretion and included regional anesthesia (nerve block), topical anesthesia (4% lidocaine cream or EMLA eutectic mixture of lidocaine 2.5% and prilocaine 2.5%), and ice and cool packs.

### Injection Technique

Treating physicians aimed for intradermal injection of the product by using blanching serial puncture, linear threading or cross-hatching, raising a wheal with a 30-gauge ½-inch needle bevel-up at an angle of 5 to 15° to the skin surface. During linear threading or cross-hatching, investigators aimed to keep the needle superficial enough to be visible through the skin surface. Injections could be anterograde or retrograde, and postinjection skin massage was permitted.

### Study End Points and Variables

#### Safety End Points

Subjects assessed their injection site pain after each injection on a 100-mm visual analog scale (VAS). For 14 days after each treatment visit, subjects recorded the nature, severity, and duration of any local injection site events in a 14-day common treatment responses (CTRs) diary. Any CTR persisting at the 14-day timepoint was automatically considered an AE. Periodic safety evaluations included CTR and AE review and lip functionality assessment and were conducted on-site at Weeks 2, 4, 8, 12, 16, 24, 36, and 52, as well as 4 weeks after Week 52 retreatment if performed.

#### Efficacy End Points

The primary study end point was the severity score for perioral rhytides on the PR-SRS, as assessed by the BLE at Week 8 after the last treatment (initial or touch-up). Of note, the PR-SRS is a photo-validated scale. Repeatability and reproducibility of subject grading using the scale was confirmed by statistical analysis of the validation data (weighted kappa scores for intrarater and interrater agreement were all >0.7; data not published).

Secondary end points included the Perioral Rhytids Domain of the FACE-Q, a validated patient-reported outcome measure^16–18^; BLE assessment of improvement on the Global Aesthetic Improvement Scale (GAIS); and subject satisfaction.

Efficacy evaluations were conducted by the BLE at Weeks 8, 12, 16, 24, 36, and 52 after the last treatment. Subject self-evaluations were also performed at Weeks 2 and 4. Efficacy end points were assessed separately for the treatment and control groups and compared up to Week 8; they were then evaluated within the pooled population up to Week 52 after last treatment.

Exploratory end points assessed in the pooled population until Week 52 included the following: BLE grading on the PR-SRS and GAIS; Subject Satisfaction; Natural Look & Feel score; and the Perioral Rhytids Domain of the FACE-Q. Subject satisfaction with the study treatment was evaluated using a 5-point scale from very satisfied (Grade 1) to very dissatisfied (Grade 5). Natural Look & Feel was assessed on a study-specific, 11-point scale ranging from 0 (unnatural) to 10 (natural); any subject with a score of ≥7 was deemed a responder for this assessment.

### Statistical Analysis

The primary objective of the study was to demonstrate statistical superiority of RHA_R_ filler to no-treatment, based on improvement of perioral rhytides as assessed by the BLE on the PR-SRS at Week 8.

The primary end point was a co-primary end point that required 3 conditions to be met: the responder rate in the RHA_R_ treatment group had to be (1) statistically superior, and (2) ≥50 percentage points higher compared with no-treatment control; and (3) above 70%. Responders were defined as subjects achieving ≥1-point improvement on the PR-SRS.

Per protocol (PP), intent-to-treat (ITT), and safety (SAFT) populations were defined for statistical analysis. The PP population consisted of subjects who had completed 8 weeks of treatment follow-up without any major protocol deviation. The ITT population included all randomized subjects who had received at least one treatment with RHA_R_ filler in compliance with their treatment protocol allocation (randomized group assignment). The SAFT population comprised all subjects who had received at least one treatment, whether or not they complied with their treatment protocol allocation.

Analyses of the primary and secondary end points of efficacy were performed separately for the ITT and PP populations. Safety descriptive analyses used the SAFT population.

One-sided Fisher exact tests were used to detect significant differences between the treatment and control groups for the primary and secondary end points, with a 0.025 significance level.

## Results

### Study Population

Two hundred two subjects with moderate-to-severe perioral rhytides (PR-SRS Grade 2 or 3) were enrolled at 8 study sites, 6 in the United States and 2 in Canada. One hundred fifty subjects were randomized to the treatment group and 52 to the control group. The sizes and dispositions of the PP, ITT, and SAFT populations are detailed in **Supplemental Digital Content 2** (see Figure S2, http://links.lww.com/DSS/A912, consort diagram). The mean ages in the treatment group and control group were 61.6 ± 7.2 years and 60.7 ± 7.6 years, respectively. Ninety-eight percent of the subjects were women and 27% had Fitzpatrick skin Phototypes IV to VI. There were 35 (17.3%) Hispanic, 4 (2.0%) African American, and 2 (1.0%) Asian subjects (see **Supplemental Digital Content 3**, Table S1, http://links.lww.com/DSS/A914, which demonstrates subject demographics and injection volume).

### Injection Volume and Technique

The mean initial injection volume of RHA_R_ was 2.0 ± 1.2 mL. At Week 2, 68% of subjects received a touch-up treatment with an average injected volume of 1.2 ± 1.1 mL. The total volume—including initial and touch-up treatments—to achieve optimal correction of perioral rhytides was 2.8 ± 2.0 mL.

Nine subjects who had perioral furrows and were on the extreme end of Grade 3 (severe) of the PR-SRS received significantly higher volumes of RHA_R_ (≥5 mL). When excluding these outliers, the mean initial and touch-up volumes of RHA_R_ were 1.8 ± 1.0 mL and 1.1 ± 0.9 mL, respectively, and the total volume required for optimal correction was 2.5 ± 1.6 mL.

Early repeat treatments at Week 12, 16, 24, or 36 occurred in a total of 17.6% of subjects—3.3%, 2.2%, 4.7% and 8.5%, respectively. The mean injected volume for those early repeat treatments was approximately 2 mL.

Taking all injections into account (initial, touch-up, early repeat treatment, and final retreatment), mean total injected volume ranged from 1.4 to 7.7 mL over the 52-week study period.

### Primary Effectiveness

**Figure 2.**
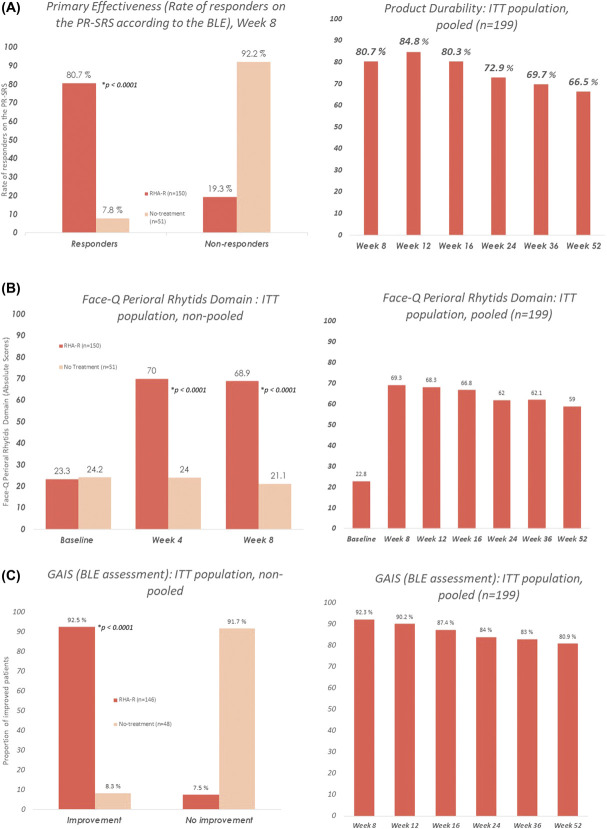
Effectiveness end points at Week 8 in the treatment group versus no-treatment control, and throughout the study in the pooled population.

The primary efficacy end point at Week 8 was achieved. Resilient Hyaluronic Acid Redensity showed statistically significant superiority to no-treatment for the correction of dynamic perioral rhytides, as assessed on the PR-SRS (80.7% vs 7.8% responder rate, *p* < .0001) (Figure 2A). The 2 co-primary end points were met, as the responder rate of the treatment group was above 70%, and more than 50 percentage points higher than that of the control group.

The effect was durable, with 66% subjects of the pooled population still classified as responders at Week 52 (Figure 2A).

**Figure 3.**
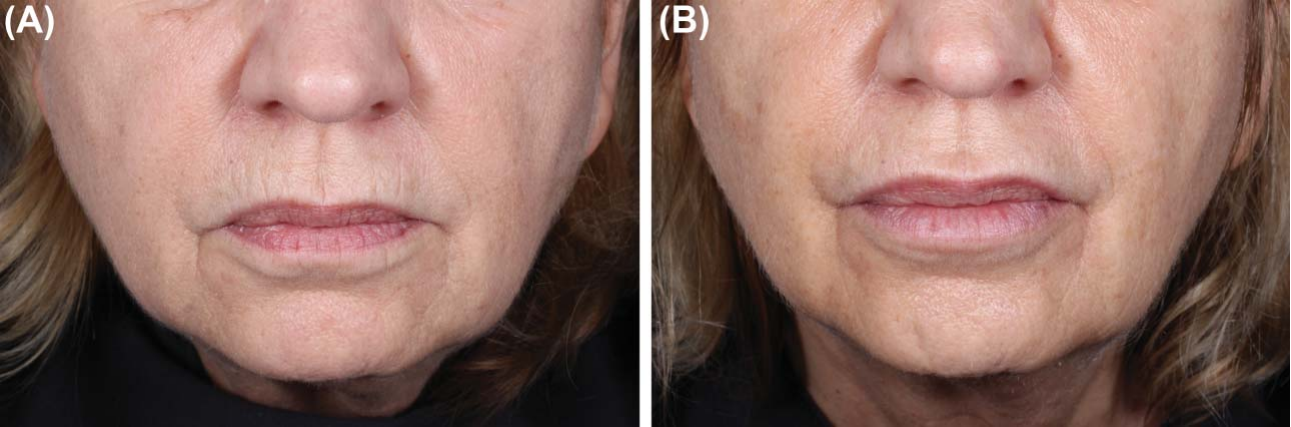
Before (A) and after (B) photographs of a 69-year-old female subject with moderate perioral rhytides at baseline (A), who improved to mild at Week 8 posttreatment (B). Initial volumes injected into the upper and lower perioral rhytides were, respectively, 0.6 and 0.4 mL, with touch-up volumes of 0.5 and 0.4 mL. Injection techniques were retrograde linear threading and serial punctures.

Representative pretreatment and posttreatment subject images are shown in Figure 3.

### Secondary and Exploratory End Points

#### FACE-Q

Assessment of patient-reported outcomes with the FACE-Q showed significantly higher scores at Weeks 4 and 8 for the treatment group than for the control group. FACE-Q scores were maintained in the pooled population after Week 8, with a mean of 59 points at Week 52 (Figure 2b).

#### Global Aesthetic Improvement Scale

As assessed by the BLE using the GAIS, the degree of aesthetic improvement at Week 8 was significantly higher for the treatment group than for the control group (92.5% vs 8.3%, *p* < .0001) (Figure 2C). After Week 8, the GAIS was assessed by the BLE at each subsequent visit until Week 52 for the pooled population. Improvement was still visible in 80.9% of subjects at their last visit.

#### Subject Satisfaction

The proportion of subjects satisfied with treatment peaked at 91.8% at Week 4 and was 88.3% at Week 52 (see **Supplemental Digital Content 4**, Figure S3, http://links.lww.com/DSS/A913, which demonstrates subject satisfaction).

#### Natural Look & Feel Scale

The percentage of subjects who scored their natural look and feel as ≥7/10 was 46% before treatment, 88.8% at Week 8 after treatment, and 87.2% in the pooled population at Week 52.

#### Safety

Adverse events were recorded by the treating investigator at study visits. Subjects also completed a daily diary, recording the presence, duration, and severity of predefined CTRs for 14 days after each treatment. Any CTR that was ongoing on the last diary day was automatically considered an AE. There were no serious treatment-related AEs (TRAEs), unanticipated device-related events or late-onset TRAEs, nodular complications, or vascular compromise events. Subjects experiencing at least one TRAE accounted for 31.1% of the SAFT population. All TRAEs were mild to moderate in severity, all resolved, and none was deemed clinically significant.

Three TRAEs (1.6%) were reported independently of the CTR diary: skin discoloration, headache, and oral herpes simplex.

The rate of CTRs after initial injection was comparable with the rate after touch-up. Across all injections performed (initial, touch-up, and early repeat treatment), the most common TRAEs were lumps and bumps/contour irregularities (17%), firmness (11.0%), and injection site bruising (10.5%) (see **Supplemental Digital Content 5**, Table S2, http://links.lww.com/DSS/A915, which demonstrates the CTR rates after initial injection).

Twenty-six (14.0%) TRAEs originated from a CTR diary section labelled “Other,” where symptoms could be written in. They included headache, hypoesthesia, injection site paresthesia, and injection site scabbing.

Mean score on the 100-mm VAS for pain was 19.9 mm (95% confidence interval [CI] [17.1–22.6]) during initial injection and 3.1 mm (95% CI [2.0–4.3]) 15 minutes after injection. Assessments of lip functionality were unchanged postinjection, except for one subject who reported moderate inability to pucker while whistling, which resolved within 1 day.

Thirty-seven percent of subjects with Fitzpatrick skin phototypes I to III experienced at least one TRAE, as compared with 35.2% of subjects with photoypes IV to VI.

## Discussion

This publication reports the first randomized, no-treatment control clinical trial to evaluate efficacy, safety, tolerability, and durability of Resilient Hyaluronic Acid Redensity (RHA_R_), a novel RHA dermal filler, for improvement of perioral rhytides. A large study cohort inclusive of all Fitzpatrick skin phototypes provides evidence to support the indication for RHA_R_ in the diverse North American population.

Resilient Hyaluronic Acid Redensity was superior to no-treatment (*p* < .0001) for correction of moderate-to-severe dynamic perioral rhytides, as rated by BLEs on a proprietary wrinkle severity scale (PR-SRS). Scores on GAIS and patient satisfaction scales were consistently high. Treatment outcomes were durable through Week 52, with a small proportion (17.6%) of subjects requiring early retreatment.

Based on AEs monitored throughout the study, treatment with RHA_R_ was safe and well-tolerated. Most TRAEs were CTR that were documented as AEs due to their persistence on or beyond the last day of the subjects' 14-day postinjection diaries. The reporting of lumps and bumps typically reflected the subjects' ability to feel some product under the skin due to its superficial placement, which resolved spontaneously. No late-onset nodules were noted.

Previous studies have attributed lesser durability in mobile facial areas to frequent muscular activity, resulting in shearing forces that promote filler degradation.^19,20^ In the perioral region, the desire for durability must be weighed against the risk of nodules from filler products that are too firm or subject to displacement within the tissue due to strong muscular forces. In this study, RHA_R_ provided lasting aesthetic improvement without emergence of late-onset AEs throughout the study evaluation period of 52 weeks.

The results of this study agree with prior randomized controlled trials that have demonstrated the safety, efficacy, and lasting effects of other RHA fillers for aesthetic treatments in dynamic facial areas.^21,22^

Study limitations included the participation of only 4 male subjects, representing 2% of the study population. Additionally, only 3 subjects had Fitzpatrick skin phototype VI; all were enrolled in the RHA_R_ treatment group. One challenge to recruitment of subjects with skin phototype VI and of male patients was the inclusion criterion of moderate-to-severe perioral rhytides. Both higher phototype individuals and male patients have a lower incidence of perioral rhytides.^23^ This challenge may be addressed in the future studies through expanded inclusion criteria and/or a specific focus on underrepresented patients. Nevertheless, no significant difference in safety outcomes was found between lower (I–III) and higher (IV–VI) phototypes in this study; the treatment was well tolerated by all subjects.

Injection volumes were variable between subjects, as shown by large standard deviations, and some were higher than expected for superficial fine lines. This can be explained by the inclusion of subjects with significant perioral furrows, who were at the extreme end of PR-SRS Grade 3 (“severe”), thereby needing greater volumes to achieve an optimal correction.

It has been saliently noted that clinical studies of fillers differ in a number of respects from everyday dermatological practice.^24^ This reflects the limitations of clinical trials whose efficacy end points must be selected to show effects of a single product. In everyday clinical practice, sustained perioral correction may be more volume-efficient because dermal fillers can be combined with botulinum toxin treatment.

## Conclusion

In this prospective, multicenter, blinded-evaluator controlled study of 202 subjects of all Fitzpatrick skin phototypes, RHA_R_ was shown to be effective and safe for the correction of dynamic perioral rhytides.

Resilient Hyaluronic Acid Redensity demonstrated marked durability, with 66% of subjects maintaining a clinical response at the Week 52 visit. Importantly, no late-onset AEs were observed. Aesthetic improvement assessed on the GAIS was maintained throughout the study, and patient satisfaction was consistently high.

Resilient Hyaluronic Acid Redensity is the most superficially implanted product in a range of RHA fillers developed to treat wrinkles and folds in different areas while accommodating facial dynamics. This range of RHA fillers that provides durable results with a low degree of crosslinking modification of the HA chains may represent a novel and useful addition to the aesthetic toolbox.

## Supplementary Material

SUPPLEMENTARY MATERIAL
