# Supplementary figures and images for: Efficacy and Safety of a New Resilient Hyaluronic Acid Filler in the Correction of Moderate-to-Severe Dynamic Perioral Rhytides: A 52-Week Prospective, Multicenter, Controlled, Randomized, Evaluator-Blinded Study
Source: Dermatol Surg. 2021 Sep 30;48(1):87–93. doi: 10.1097/DSS.0000000000003238 (PMC8667798; doi:10.1097/DSS.0000000000003238)

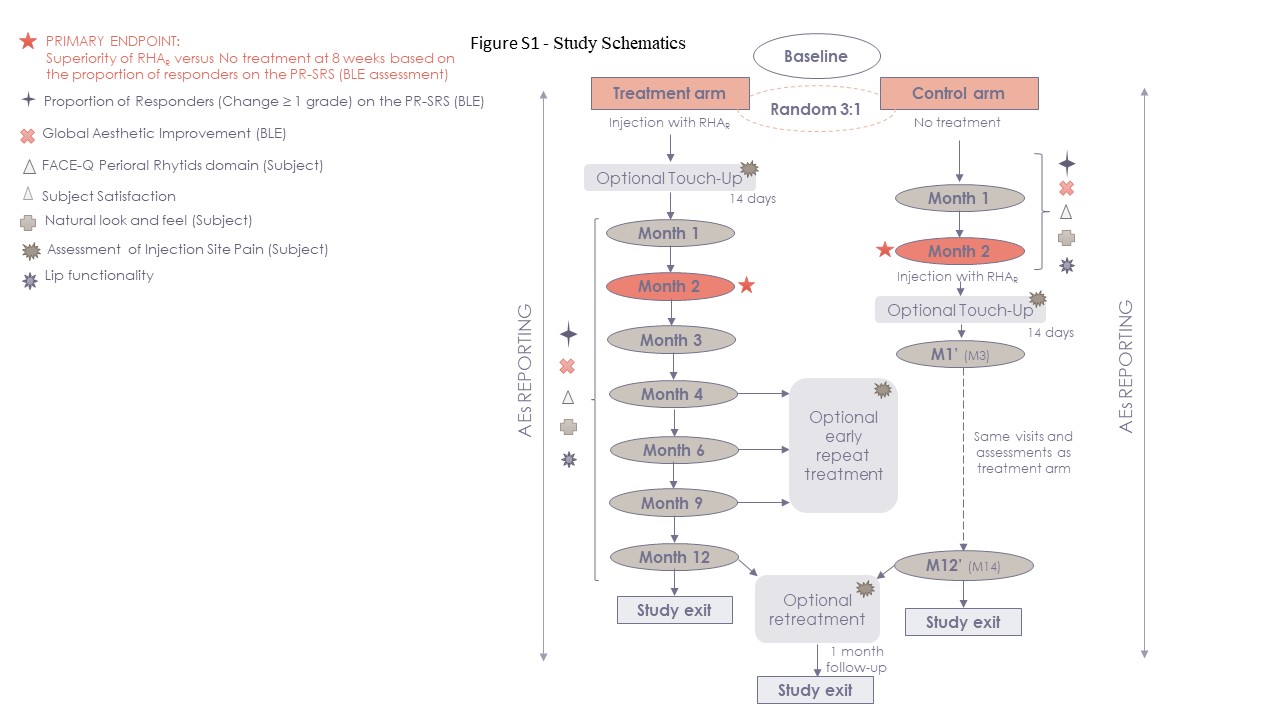

Supplement: SUPPLEMENTARY MATERIAL [file ds-48-087-s001.jpg]

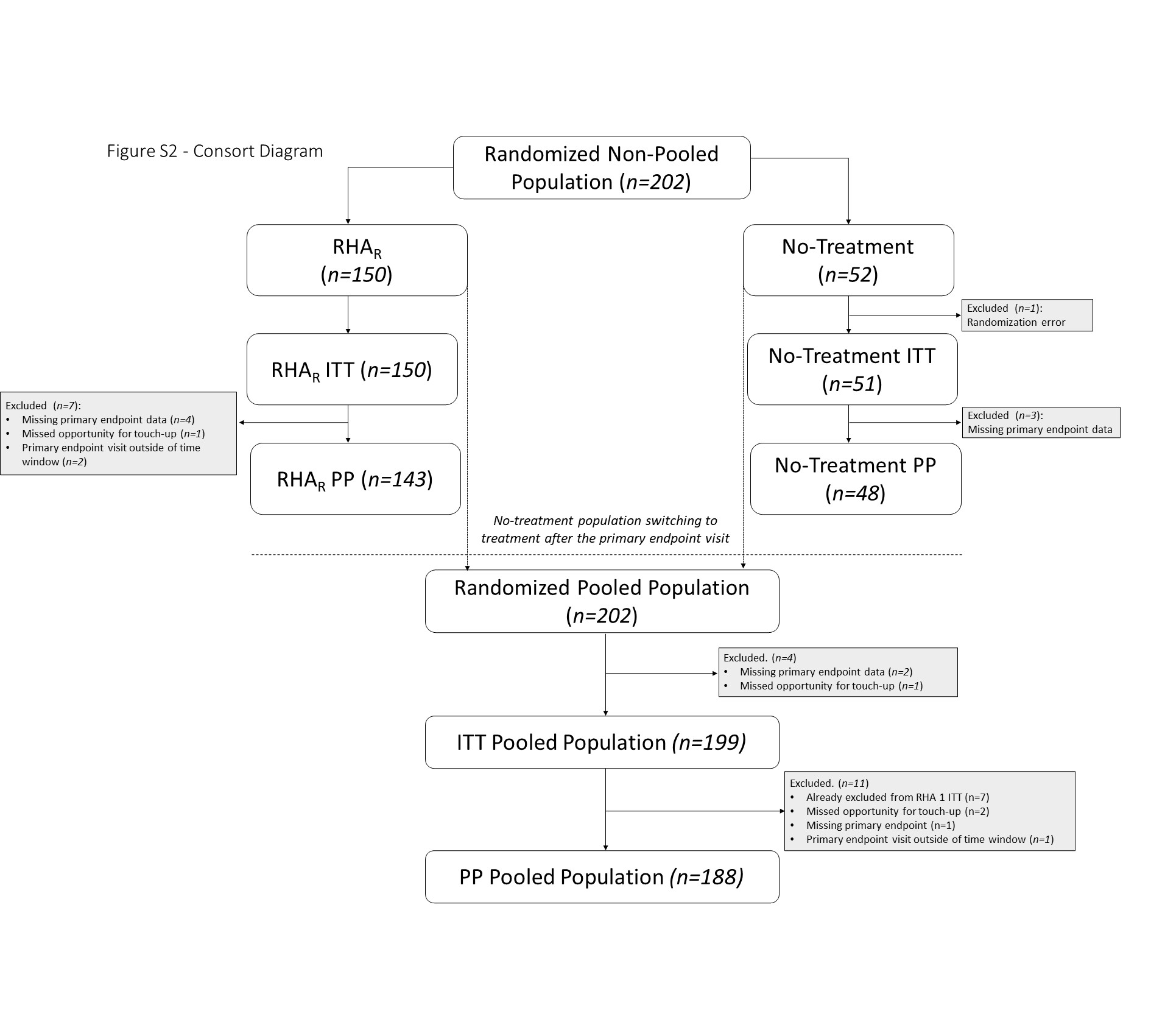

Supplement: SUPPLEMENTARY MATERIAL [file ds-48-087-s002.jpg]

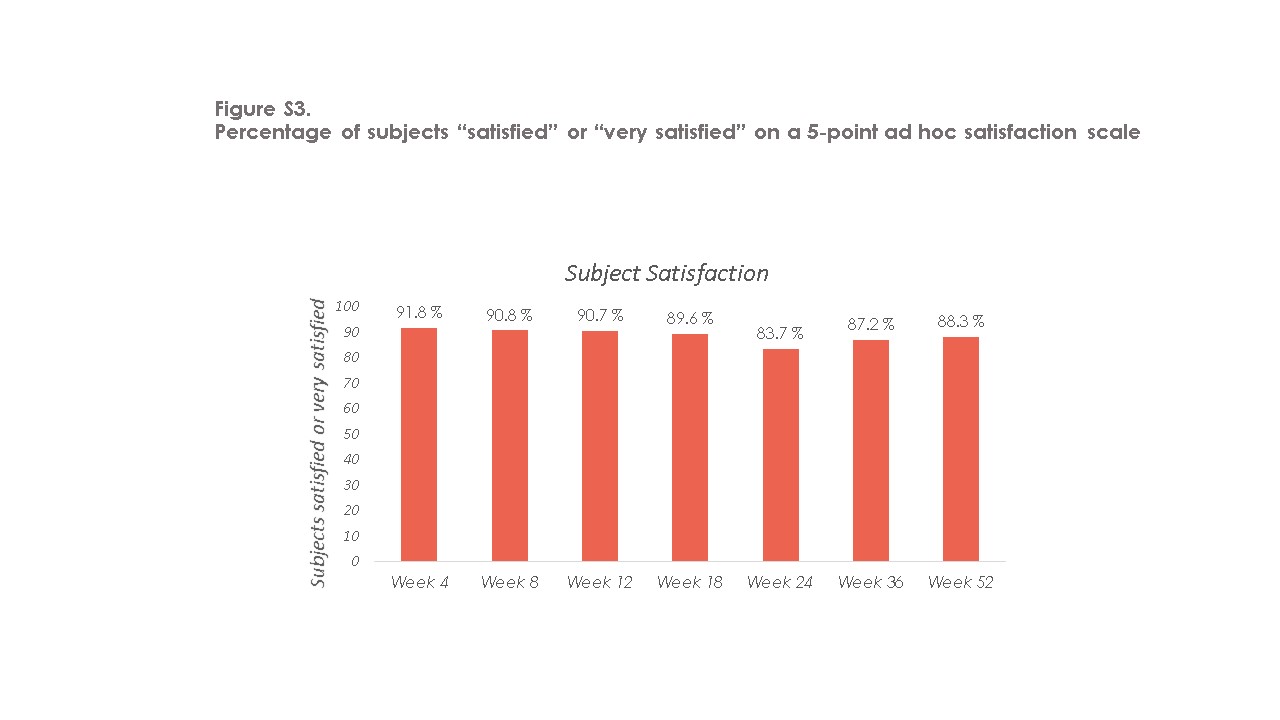

Supplement: SUPPLEMENTARY MATERIAL [file ds-48-087-s003.jpg]
