# Supplementary material for: Efficacy and Safety of a New Resilient Hyaluronic Acid Filler in the Correction of Moderate-to-Severe Dynamic Perioral Rhytides: A 52-Week Prospective, Multicenter, Controlled, Randomized, Evaluator-Blinded Study
Source: Dermatol Surg. 2021 Sep 30;48(1):87–93. doi: 10.1097/DSS.0000000000003238 (PMC8667798; doi:10.1097/DSS.0000000000003238)
Supplement: SUPPLEMENTARY MATERIAL [file ds-48-087-s004.docx]

**Table S1 Subject Demographics and Injection Volume**

| **Variable** | **RHA_R_ Group (N=150)** | **No-Treatment Control Group (N=52)** |
| --- | --- | --- |
| **Age, years** | 61.6 ± 7.2 | 60.7 ± 7.6 |
| **Female, n (%)** | 147 (98.0%) | 51 (98.1%) |
| **Race, n (%)** | | |
| White | 143 (95.3%) | 52 (100.0%) |
| Black or African American | 4 (2.7%) | 0 (0.00%) |
| American Indian or Alaska Native | 1 (0.7%) | 0 (0.00%) |
| Asian | 2 (1.3%) | 0 (0.00%) |
| **Ethnicity, n (%)** | | |
| Hispanic or Latin American | 25 (16.7%) | 10 (19.2%) |
| Not Hispanic or Latin American | 125 (83.3%) | 42 (80.8%) |
| **Fitzpatrick Skin Phototype** | | |
| **I-III** | **110 (73.3%)** | **37 (71.2%)** |
| I | 18 (12.0%) | 6 (11.5%) |
| II | 37 (24.7%) | 13 (25.0%) |
| III | 55 (36.7%) | 18 (34.6%) |
| **IV-VI** | **40 (26.7%)** | **15 (28.8%)** |
| IV | 29 (19.3%) | 12 (23.1%) |
| V | 8 (5.3%) | 3 (5.8%) |
| VI | 3 (2.0%) | 0 (0.0%) |
| ***RHA_R_ Mean Volume Injected*** | | *Note: Control Group received treatment with RHA_R_ at Week 8* |
| Initial injection (mean ± SD) | 1.9 ± 1.2 mL | 2.2 ± 1.3 mL |
| Touch-up | 1.2 ± 1.1 mL | 1.2 ± 1.0 mL |
| Total | 2.8 ± 2.0 mL | 2.9 ± 1.7 mL |
