# Supplementary material for: Efficacy and Safety of a New Resilient Hyaluronic Acid Filler in the Correction of Moderate-to-Severe Dynamic Perioral Rhytides: A 52-Week Prospective, Multicenter, Controlled, Randomized, Evaluator-Blinded Study
Source: Dermatol Surg. 2021 Sep 30;48(1):87–93. doi: 10.1097/DSS.0000000000003238 (PMC8667798; doi:10.1097/DSS.0000000000003238)
Supplement: SUPPLEMENTARY MATERIAL [file ds-48-087-s005.docx]

**Table S2 CTR by Severity After Initial Injection (Safety Population)**

| **Common Treatment Responses** | **RHA_R_ (N=199 Subjects) n/%** | | | | |
| --- | --- | --- | --- | --- | --- |
|  | **≥ 1 CTR** | **None** | **Mild** | **Moderate** | **Severe** |
| **Redness** | 131 (65.8%) | 68 (34.2%) | 84 (42.2%) | 42 (21.1%) | 5 (2.5%) |
| **Pain** | 54 (27.1%) | 145 (72.9%) | 39 (19.6%) | 13 (6.5%) | 2 (1.0%) |
| **Tenderness** | 105 (52.8%) | 94 (47.2%) | 83 (41.7%) | 19 (9.5%) | 3 (1.5%) |
| **Firmness** | 115 (57.8%) | 84 (42.2%) | 79 (39.7%) | 33 (16.6%) | 3 (1.5%) |
| **Swelling** | 146 (73.4%) | 53 (26.6%) | 85 (42.7%) | 49 (24.6%) | 12 (6.0%) |
| **Lumps/bumps** | 115 (57.8%) | 84 (42.2%) | 71 (35.7%) | 34 (17.1%) | 10 (5.0%) |
| **Bruising** | 154 (77.4%) | 45 (22.6%) | 65 (32.7%) | 65 (32.7%) | 24 (12.1%) |
| **Itching** | 31 (15.6%) | 168 (84.4%) | 26 (13.1%) | 3 (1.5%) | 2 (1.0%) |
| **Discoloration** | 94 (47.2%) | 105 (52.8%) | 49 (24.6%) | 34 (17.1%) | 11 (5.5%) |
